# Response to treatment with an ALK-TKI in a patient with advanced lung adenocarcinoma with concurrent ALK fusion and high PD-L1 expression: A case report

**DOI:** 10.1097/MD.0000000000030094

**Published:** 2022-08-19

**Authors:** Yaping Zhang, Hongming Fang, Jianfeng Hong, Xiaoyan Wang, Hui Wang, Guoqiang Pan

**Affiliations:** a Xiaoshan Hospital, Hangzhou Normal University, Hangzhou, China; b Hangzhou Normal University, Hangzhou, China.

**Keywords:** ALK, nonsmall cell lung cancer, PD-L1

## Abstract

**Rationale:**

Previous studies have shown that PD-L1 TPS ≥50% in lung cancer rarely overlaps with driver oncogenes such as epidermal growth factor receptor and anaplastic lymphoma kinase (ALK). The initial gene detection of the patient in this study showed ALK fusion combined with high expression of PD-L1. We explored the treatment options for this patient.

**Patient concerns:**

A 34-year-old woman presented for the first time with “repeated fever and cough for 20 days.” The patient denied any underlying medical history.

**Diagnosis:**

After a series of imaging examinations and needle biopsy, the patient was diagnosed as stage IV lung adenocarcinoma with multiple liver and bone metastases (EML4-ALK fusion, PD-L1 TPS 80%).

**Interventions:**

The patient was initially given alectinib targeted therapy. After progression, a second round of genetic testing was performed and the patient was detected to have both ALK fusion and BRAF mutation. The patient was then successively changed to treatment with ensatinib combined with dabrafenib, and lorlatinib combined with dabrafenib.

**Outcomes:**

The initial efficacy evaluation of alectinib was PR, but its PFS was only 4 months. The patient only achieved an overall survival of 10 months.

**Lessons:**

Non–small cell lung cancer with an ALK fusion and high PD-L1 expression responds poorly to most current treatment options, with survival time after ALK-tyrosine kinase inhibitor treatment notably shorter than that of patients with an ALK fusion alone.

## 1. Introduction

Recent years have seen many advances in both the diagnosis and treatment of lung cancer. Among them, the progresses of driver gene-related targeted therapy and immunotherapy have been particularly encouraging. Importantly, these advances have brought the possibility of better long-term survival to patients with metastatic lung cancer. The presence of these genetic drivers is predictive of the therapeutic efficacy of corresponding oral tyrosine kinase inhibitors (TKIs). The emergence of immune checkpoint inhibitors (ICIs) has brought new hope to patients lacking driver gene mutations. Previous studies have shown that PD-L1 TPS ≥50% rarely overlaps with the presence of epidermal growth factor receptor (EGFR), anaplastic lymphoma kinase (ALK), c-ros oncogene 1 (ROS1), and other driving oncogenes,^[1]^ however, herein we report a patient whose initial genetic testing showed an ALK fusion combined with high PD-L1 expression. What should be the priority choice of treatment?

## 2. Clinical information

The patient (female, 34 years old) was admitted to the respiratory department of our hospital in April 2021 because of “repeated fever and cough for 20 days.” The patient had an ECOG score of 1. The patient had no underlying medical history smoking or alcoholism history, as well as family history of cancer.

**Figure 1.**
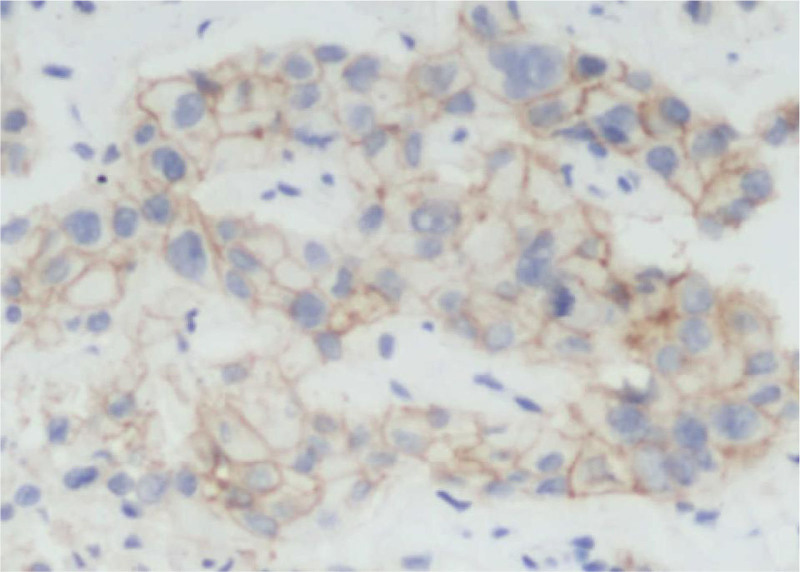
PD-L1 expression report for April 2021.

**Figure 2.**
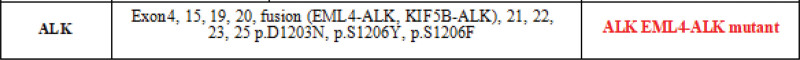
Genetic test report for April 2021.

In April 2021, the patient’s chest CT showed “multiple nodules in both lungs, multiple enlarged lymph nodes in the mediastinum and hilum; attached to the right lobe of the liver with a slightly low-density mass shadow” (Fig. 4A, B) and an abdominal CT showed “multiple mass of liver” (Fig. 4C). Bone scan showed “abnormal enhancement of bone metabolism in multiple parts of the body”. On April 19, 2021, a biopsy of the “right liver mass and right supraclavicular mass” was performed, and the pathological indications (right liver mass aspiration, right supraclavicular mass aspiration) were poorly differentiated adenocarcinoma. Combined with the results of immunohistochemistry, the first consideration was lung adenocarcinoma metastasis. The final diagnosis was “stage IV lung adenocarcinoma with multiple liver and bone metastases.” Genetic testing showed “EML 4-ALK fusion, PD-L1 TPS 80%” (Figs. 1 and 2).

**Figure 3.**
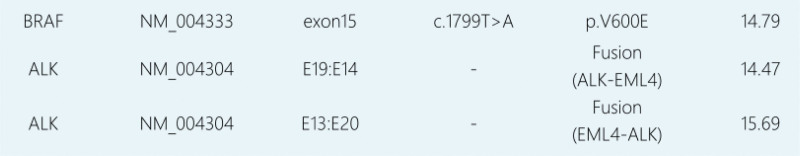
Genetic test report for September 2021.

**Figure 4.**
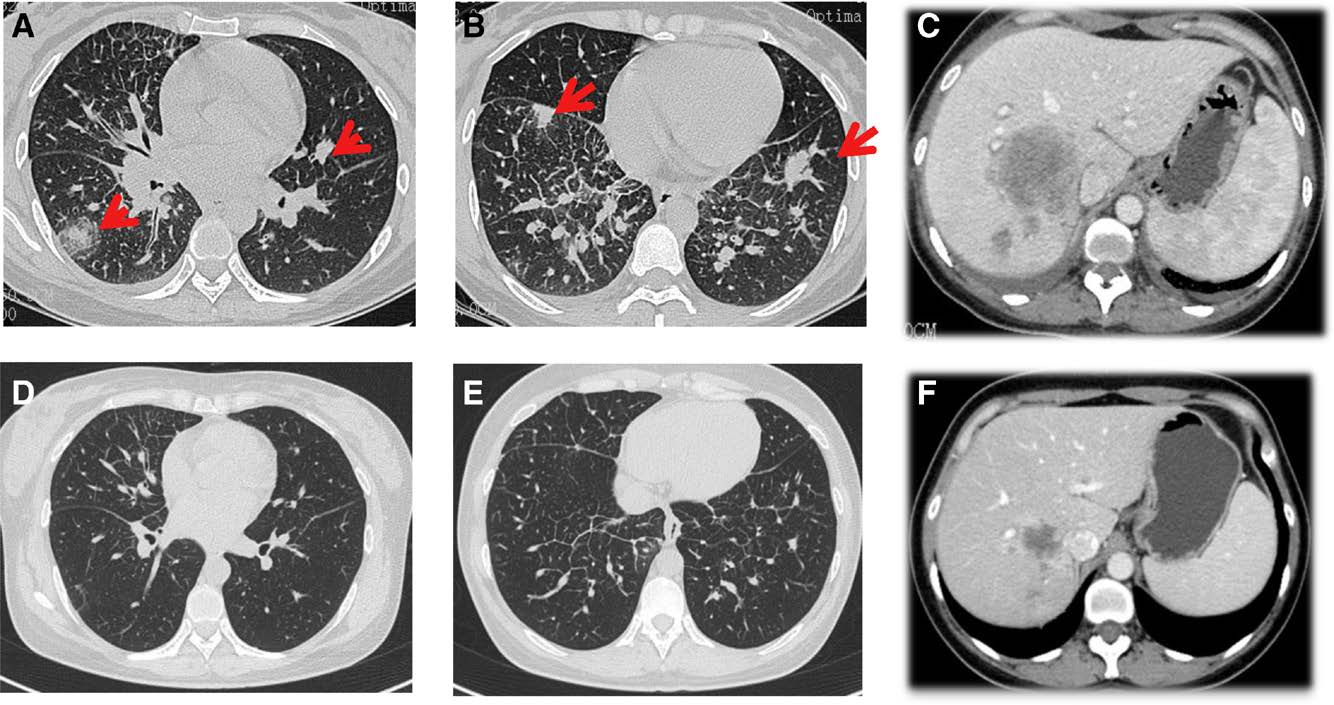
CT scans for (A–C) April 2021 and (D–F) June 2021. CT = computed tomography.

**Figure 5.**
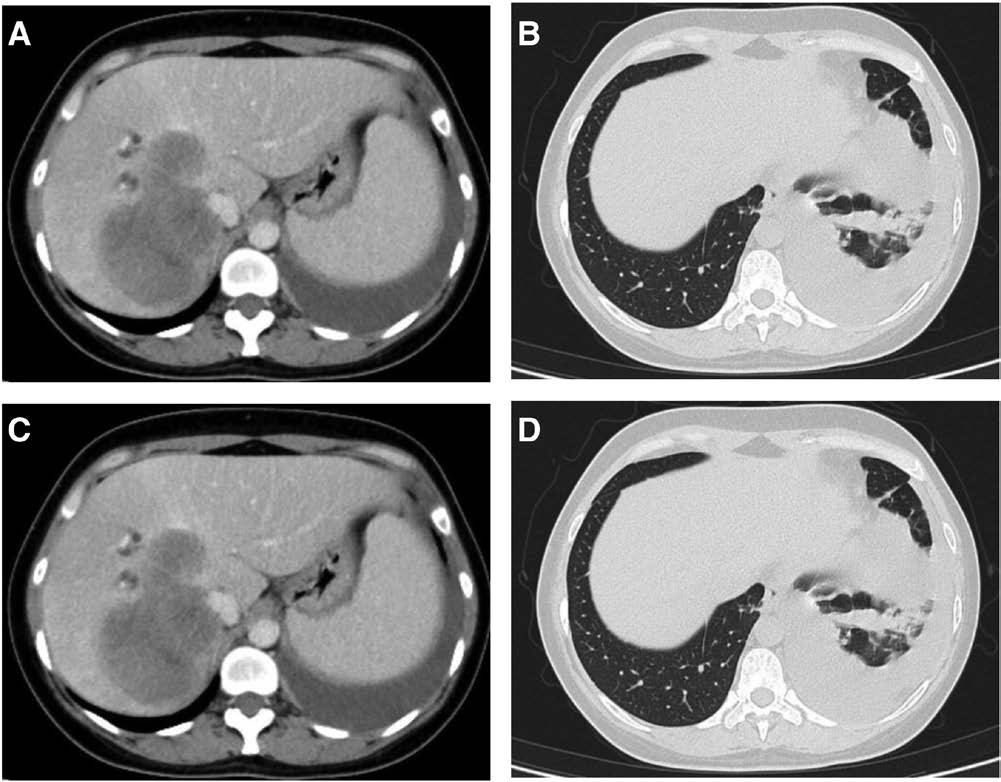
CT scans for (A and B) June 2021 and (C and D) August 2021. CT = computed tomography.

On May 1, 2021, the patient started oral alectinib targeted therapy, and reexamination on June 25, 2021, using PR to determine treatment efficacy (according to RECIST1.1) showed that the lung lesions and liver metastases were significantly reduced (Fig. 3D–F). Reexamination on August 27, 2021 using PD to determine treatment efficacy (according to RECIST1.1) showed that the liver lesions became larger than before (Fig. 4A–D). A second liver biopsy was performed, and subsequent pathology showed “(liver) poorly differentiated carcinoma, and lung adenocarcinoma metastasis was considered in combination with immunohistochemistry analysis.” Tissue biopsy was subjected to a second round of genetic testing, which indicated “BRAFV600E 14.79%, EML 4-ALK fusion 14.47%” (Fig. 5). On September 20, 2021, the patient’s treatment was changed to a combination of ensatinib with dabrafenib targeted therapy. After treatment began, the patient experienced obvious fever, chills, fatigue, and anorexia. During this period, the patient developed a large amount of pleural effusion on the left side, and the exfoliated cells in the pleural effusion were “atypical cells with consideration of adenocarcinoma.” The patient was subsequently administered 1 intrapleural infusion of platinum.

**Figure 6.**
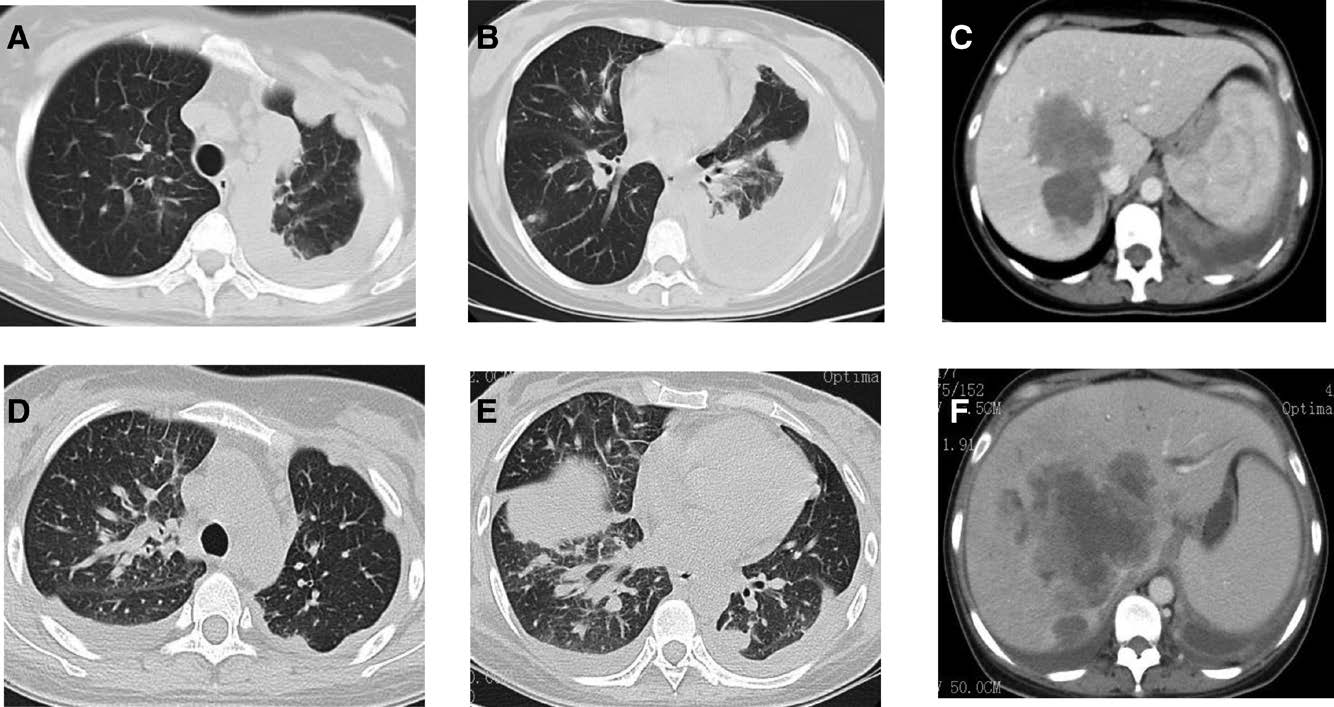
CT scans for (A–C) November 2021 and (D–F) January 2022. CT = computed tomography.

One month later, reexamination showed that the lung and intrahepatic lesions had progressed (Fig. 6A–C) (according to RECIST1.1), and her antitumor treatment was changed to 1 treatment cycle of “pemtrexed add carboplatin and bevacizumab.” From December 2021 to January 2022, the patient’s treatment was again changed, this time to “lorlatinib combined with dabrafenib” targeted therapy. Efficacy was assessed using PD combined with chest and abdomen CT (Fig. 6D–F) (according to RECIST1.1). The patient was unable to tolerate chemotherapy, targeted therapy, IO therapy, or any other active antitumor therapy. This was because the general condition of the patient was poor, including symptoms like fever, abdominal pain, and elevated bilirubin levels. Subsequently, the patient was given symptomatic and supportive treatment including intravenous nutrition and analgesia. The patient passed away on February 4, 2022, and the overall survival (OS) was only 10 months.

## 3. Discussion

According to the statistical analysis of global cancer data in 2020, there were an estimated 2.2 million new cancer cases and 1.8 million deaths. Of these, lung cancer was the second most commonly diagnosed cancer and the leading cause of cancer death.^[2]^ Non–small cell lung cancer (NSCLC) accounts for >80% of all lung cancer cases and adenocarcinoma is the most common type.^[3]^ Critically, approximately 50% of patients with NSCLC had already metastasized at the time of diagnosis. This results in a poor prognosis for these patents.

According to previous reports, the 5-year relative survival rate of patients with NSCLC is only 5.5%.^[4]^ Despite this, recent years have seen many advances in both the diagnosis and treatment of lung cancer. Among them, the progress of driver gene-related targeted therapy and immunotherapy has been particularly encouraging. Importantly, these advances have brought the possibility of better long-term survival to patients with metastatic lung cancer.

In patients with advanced NSCLC, the mutation rate of driver genes is as high as 60%. Common driver genes include EGFR, ALK, ROS1, Kirsten rat sarcoma viral oncogene homolog (KRAS), and human epidermal growth factor receptor-2.^[5]^ The presence of these genetic drivers is predictive of the therapeutic efficacy of corresponding oral TKIs, which are associated with more durable outcomes, less toxicity, and a better quality of life compared with conventional chemotherapy.^[6]^ Among these, the benefit of TKI therapy in tumors with an ALK mutation is particularly significant. As a result, it is known as a “diamond mutation.” In 2007, the EML4-ALK fusion type was first discovered in NSCLC and confirmed to be a driver gene in lung cancer.^[7]^ The incidence of ALK fusion-positive NSCLC is about 5%, with no significant difference between Eastern and Western populations.^[8]^ ALK-TKIs, such as alectinib, crizotinib, and lorlatinib, have been shown to be significantly more effective than chemotherapy.^[8–10]^

The emergence of ICIs has brought new hope to patients lacking driver gene mutations. ICIs either alone or in combination with chemotherapy have been shown to significantly prolong the OS time of lung cancer patients. More specifically, the 5-year survival rate of patients with advanced NSCLC treated with ICIs has been shown to exceed 15%.^[11,12]^Although predictive biomarkers of ICIs remain unknown, PD-L1 expression in both tumor and immune cells has been reported to correlate with treatment outcomes in NSCLC patients.^[13,14]^

In the case study presented here, initial genetic testing showed that there was an ALK fusion combined with high PD-L1 expression. The bigger question remains: Is this a coincidence or is there a relationship between the expressions of the 2? Previous studies have shown that PD-L1 TPS ≥50% rarely overlaps with the presence of EGFR, ALK, ROS1, and other driving oncogenes.^[1]^ However, Ma et al^[15]^ showed that the expression of PD-L1 protein in a human lung adenocarcinoma cell line with EML4-ALK fusion gene was higher than that in an identical cell line without the fusion gene. Moreover, PD-L1 protein expression was significantly increased when EML4-ALK expression was induced in human lung adenocarcinoma cells. To this end, Yoneshima et al^[16]^ conducted a retrospective analysis of 80 patients with EGFR/ALK-positive lung adenocarcinoma. The following breakdown was observed: 9 cases had an ALK rearrangement; PD-L1 TPS ≥1% accounted for 5 cases, of which 2 patients had TPS ≥50%, accounting for 22.2%. Koh et al^[17]^ used immunohistochemistry to evaluate PD-L1 expression in 532 lung adenocarcinomas, including 58 ALK translocation tumors. PD-L1 was detected in 47 (81%) of 58 ALK translocation patients. In patients where PD-L1 expression was determined to be moderate to strong, the proportion of patients with PD-L1 expression was as high as 25.9%. This was significantly higher than that observed in lung adenocarcinomas with an EGFR mutation, with a KRAS mutation but lacking an ALK mutation, or with either an EGFR or KRAS mutation (all *P* < .005). Furthermore, researchers found that this ALK-dependent upregulation of PD-L1 expression may be mediated by signal transducer and activator of transcription 3 and hypoxia-inducible factor-1α under both normoxic and hypoxic conditions. Work by Li et al^[18]^ and other researchers^[19]^ have also suggested that patients with ALK-positive NSCLC have a high PD-L1 expression rate. That said, the frequency of PD-L1 TPS ≥50% in patients with these driver oncogenes is unknown. Collectively, current research relevant to this question remains focused on small sample sizes and further studies will be needed.

Both TKIs and ICIs are important treatments for advanced NSCLC. Most clinical studies exploring ICIs have excluded driver gene-positive patients. However, when the patients’ clinical genetic test results show both driver gene-positive and PD-L1 high expression, what should be the choice of treatment?

Li et al^[18]^ studied 68 patients with both an ALK rearrangement and positive PD-L1. Their results suggested that the median PFS was shorter in patients with high PD-L1 expression. In a subsequent multivariate analysis, PD-L1 expression (TPS ≥ 50%) remained a negative prognostic factor for the clinical efficacy of second-generation ALK-TKIs in ALK-rearranged NSCLC. Koh et al^[17]^ analyzed the correlation between PD-L1 expression and prognosis in 81 patients with an ALK mutation. Compared with patients with negative PD-L1 expression, patients with positive PD-L1 expression had shorter PFS and OS. Moreover, the PFS and OS of patients with strong PD-L1 expression were significantly shorter than those of other patients.

What about TKI combined with ICIs? In Checkmate370,^[20]^ a subgroup of ALK-positive NSCLC patients were treated with nivolumab combined with crizotinib. The cohort had planned to enroll 20 patients, but was terminated early. This was because of the 13 patients who had been enrolled in combination therapy, 5 (38%) developed severe hepatotoxicity and 2 died. There are few related studies on using ALK-TKI combined with immunotherapy, but there are similar studies in other TKIs, such as immunotherapy combined with EGFR-TKI. For instance, the Phase Ib TATTON study of osimertinib combined with durvalumab suggested the possibility of an increased incidence of interstitial lung disease.^[21]^ In another phase I study of 56 patients receiving durvalumab in combination with gefitinib, the incidence of hepatic AEs was significantly higher than previously reported with either gefitinib or durvalumab monotherapy.^[22]^ Grade 3/4 adverse events—including ALT elevation (7%), pyrexia (7%), and rash (7%)—occurred in 39% of patients in the phase Ib study of atezolizumab plus erlotinib. Of these, 18% of patients experienced adverse events that led to treatment discontinuation.^[23]^ Further development of this approach remains controversial, given the relatively high incidence of toxicity associated with the combination of TKIs and immunotherapy. Given this, using a combination of TKI and immunotherapy requires further research. There are also case reports that patients with both an EGFR/ALK mutation and high PD-L1 expression were selected for first-line bevacizumab combined with chemotherapy and radiotherapy for pulmonary lesion. Under this treatment regimen, the curative effect reached PR and was followed by ALK-TKI maintenance therapy.^[24]^ Ultimately, the patient achieved sustained remission. In general, treatment in patients with combined mutations have poorer efficacy and treatment options will need further study.

The patient presented here initially had an ALK fusion with high PD-L1 expression. Her initial PFS was only 4 months, which was in line with the short PFS reported in the literature in other patients with an ALK fusion combined with PD-L1 positivity. After alectinib treatment, the patient subsequently developed a BRAF mutation on the basis of the ALK fusion and progressed. Unfortunately, the side effects of lorlatinib combined with dabrafenib were too significant. As a result, the patient did not receive the above drugs in sufficient amounts, which may have affected the outcomes. The most regrettable thing is that this patient had repeated high fever, later jaundice, and poor PS score. As a result, she was unable to receive active antitumor therapy such as immunotherapy or combined chemotherapy/targeted therapy. Otherwise, we would be able to further verify whether patients with an ALK fusion and strong PD-L1 positivity would benefit from immunotherapy.

Although our patient had a PFS of only 4 months and an OS of only 10 months on alectinib, this case served as a reminder for the clinic that when patients are detected with ALK and PD-L1 positive, the efficacy of ALK-TKI monotherapy may be poor, and the optimal treatment mode will need further exploration.

## Author contributions

**Investigation:** Jianfeng Hong.

**Resources:** Xiaoyan Wang.

**Visualization:** Hui Wang.

**Writing – original draft:** Yaping Zhang.

**Writing – review & editing:** Guoqiang Pan, Hongming Fang.
